# User experience of applying AMSTAR 2 to appraise systematic reviews of healthcare interventions: a commentary

**DOI:** 10.1186/s12874-023-01879-8

**Published:** 2023-03-16

**Authors:** Karina Karolina De Santis, Dawid Pieper, Robert C. Lorenz, Uta Wegewitz, Waldemar Siemens, Katja Matthias

**Affiliations:** 1 Department of Prevention and Evaluation, Leibniz Institute for Prevention Research and Epidemiology – BIPS GmbH, Bremen, Germany; 2 Brandenburg Medical School Theodor Fontane (MHB), Center for Health Services Research (ZVF-BB), Brandenburg an der Havel, Germany; 3 Lise Meitner Group for Environmental Neuroscience, Max Planck Institute for Human Development, Berlin, Germany; 4 Federal Institute for Occupational Safety and Health (BAuA), Division 3 Work and Health, Berlin, Germany; 5 Faculty of Medicine, Institute for Evidence in Medicine, Medical Center - University of Freiburg, University of Freiburg, Freiburg, Germany; 6 Faculty of Electrical Engineering and Computer Science, University of Applied Sciences Stralsund, Stralsund, Germany

**Keywords:** AMSTAR 2, Systematic review (SR), Evidence appraisal, Confidence rating

## Abstract

**Background:**

‘A Measurement Tool to Assess Systematic Reviews, version 2’ (AMSTAR 2) is a validated 16-item scale designed to appraise systematic reviews (SRs) of healthcare interventions and to rate the overall confidence in their results. This commentary aims to describe the challenges with rating of the individual items and the application of AMSTAR 2 from the user perspective.

**Discussion:**

A group of six experienced users (methodologists working in different clinical fields for at least 10 years) identified and discussed the challenges in rating of each item and the general use of AMSTAR 2 to appraise SRs. A group discussion was used to develop recommendations on how users could deal with the identified challenges. We identified various challenges with the content of items 2–16 and with the derivation of the overall confidence ratings on AMSTAR 2. These challenges include the need (1) to provide additional definitions (e.g., what constitutes major deviations from SR protocol on item 2), (2) to choose a rating strategy for multiple conditions on single items (e.g., how to rate item 5 if studies were selected in duplicate, but consensus between two authors was not reported), and (3) to determine rules for deriving the confidence ratings (e.g., what items are critical for such ratings). Based on these challenges we formulated specific recommendations for items 2–16 that AMSTAR 2 users could consider before applying the tool.

**Summary:**

Our commentary adds to the existing literature by providing the first in-depth examination of the AMSTAR 2 tool from the user perspective. The identified challenges could be addressed by additional decision rules including definitions for ambiguous items and guidance for rating of complex items and derivation of confidence ratings. We recommend that a team consensus regarding such decision rules is required before appraisal procedure begins.

**Trial registration:**

Not applicable.

## Background

‘A Measurement Tool to Assess Systematic Reviews, version 2’ (AMSTAR 2) is a 16-item scale designed to assess the overall confidence in the results of systematic reviews (SRs) of healthcare interventions [1]. The tool can be used to appraise various aspects of SRs of randomized-controlled trials (RCTs), non-randomized studies of interventions (NRSIs) or both, including literature searches, study selection and coding, data reporting and synthesis as well as assessment and discussion of any biases [1]. AMSTAR 2 consists of two documents: (1) AMSTAR 2 tool with 16 items and the rating guidelines for each item (supplementary figure to [1]) and (2) AMSTAR 2 guidance document with detailed explanations of the rating guidelines (supplementary appendix 1 to [1]). The individual items are rated as YES, PARTIAL YES, NO or NO META-ANALYSIS CONDUCTED based on fulfilling the item content. The overall confidence rating in the results of SR (high, moderate, low or critically low) is assigned based on a combination of ratings on the 16 items that include seven critical and nine non-critical items [1].

AMSTAR 2 is a useful appraisal tool because it is freely accessible and it has acceptable psychometric properties, including a moderate to substantial interrater reliability for most items and acceptable convergent validity with AMSTAR [2] and the Risk of Bias in Systematic Reviews (ROBIS, [3]) [1, 4–8]. First indications from AMSTAR 2 users suggest that some challenges are encountered with the tool (for example, see [9–11]). However, so far there is no detailed description of such challenges with AMSTAR 2.

This commentary aims to describe the challenges with rating of the individual items and the application of AMSTAR 2 from the user perspective. We aim to assist the AMSTAR 2 users with applying the tool in practice (i.e., to appraise SRs of healthcare interventions). We also attempt to derive recommendations for AMSTAR 2 users that could be considered before performing the appraisals to improve the usability of the tool.

## Discussion

The work on this commentary was initiated by two authors (KM and KDS) who met for a day-long kick-off meeting in October 2019. The aim of this meeting was to discuss the challenges with AMSTAR 2 encountered by both authors who work independently from each other at two academic institutions. Subsequently, we formed a group with further colleagues (other co-authors on this paper) personally known to us from our research networks who expressed an interest in the topic. All group members are senior researchers with doctoral degrees or professors who are experienced users of AMSTAR 2 in different clinical fields and experts in research methods with the work experience of at least 10 years. All members are methodologists who published their own SRs of healthcare interventions, meta-research studies on evidence appraisal with AMSTAR 2 (or its predecessor AMSTAR), or contributed to development of guidelines for research synthesis. In addition, some group members supervise student projects and teach research methods on the undergraduate and graduate levels.

The group met six times for in-depth online group discussions that lasted approximately two hours each. The meetings took place once a month between the end of 2021 and mid 2022. One author (KM) moderated the group discussion while another author (KDS) took notes that were viewed by all group members via share screen function. We discussed each AMSTAR 2 item and identified any challenges with the tool. The final consensus regarding each identified challenge was reached during our discussions and is documented in this manuscript.

## Challenges with individual AMSTAR 2 items

### Item 1. Did the research questions and inclusion criteria for the review include the components of PICO? [1] (ratings: YES, NO)

There were no challenges with this item.

### Item 2. Did the report of the review contain an explicit statement that the review methods were established prior to the conduct of the review and did the report justify any significant deviations from the protocol? [1] (ratings: YES, PARTIAL YES, NO)

Prospectively planned and written SR protocols could reduce the risk of bias in SRs [1]. There are three challenges with this item. First, the item requires that a SR contains “an explicit statement that the review methods were established prior to the conduct of the review” and that SR authors “justify any significant deviations from the protocol” [1]. However, an explicit statement without access to the protocol is insufficient to rate the protocol’s contents and any deviations. Second, it is unclear how to rate this item if SR protocol exists but deviations are either not explained or are extensive. It is also unclear how to decide if any deviations are extensive or not. Third, existence of a protocol does not guarantee that the protocol was completed before SR commenced or, at least, before study selection and possibly data coding were completed. Thus, it is unclear how to rate the item if SR protocol was registered shortly before submission of the completed SR for peer-review unless reasons are given by SR authors.

### Item 3. Did the review authors explain their selection of the study designs for inclusion in the review? [1] (ratings: YES, NO)

The inclusion of different study designs should be justified in SRs [1]. This is especially important because AMSTAR 2 can be used to appraise SRs that include different study designs, such as RCTs, NRSI, or both [1]. There are two challenges with this item. First, the inclusion of RCTs is often not explicitly explained in SRs. Although RCTs are considered the gold standard, they may not be appropriate to evaluate the outcomes in some clinical fields (for example, see [12]). Several methodological studies with AMSTAR 2 showed that this item was rated NO in over 90% of the appraised SRs [10, 13–15]. However, some SRs explained that the highest quality of evidence in their field was available from RCTs and thus implicitly justified the inclusion of RCTs. Second, item 3 wording in the AMSTAR 2 tool is open to different interpretations and appears more conservative than in the AMSTAR 2 guidance document. Specifically, the item can be rated if SR authors provide a rationale for including specific study designs in full-text of their SR according to the AMSTAR 2 guidance document [1] while an explanation for including specific study designs is required by the AMSTAR 2 tool [1].

### Item 4. Did the review authors use a comprehensive literature search strategy? [1] (ratings: YES, PARTIAL YES, NO)

Requirements for a comprehensive literature search strategy are listed in the AMSTAR 2 tool and the AMSTAR 2 guidance document [1]. There are several challenges with this item. First, to assess the search strategy in a SR, an access to the complete search strategy is required. However, according to the item it is sufficient if only the keywords are reported. Reporting of keywords only is associated with poorer reproducibility of search results than disclosing the entire search strategy. Important aspects of the search strategy also show how the search terms were combined with Boolean operators, search timeframe, last search date and search location (e.g., in titles only). Second, some Cochrane groups have their own trial databases established from searches in several other databases [16]. If available, authors performing Cochrane SR will usually search such a single database. Thus, it is unclear how to rate this item if a single database is searched even if such a database includes trials from several other databases. Third, the search for gray literature and the search in trial registries are listed as separate requirements for the YES rating in the AMSTAR 2 tool [1] while the search in trial registries is mentioned as an example of the gray literature search in the AMSTAR 2 guidance document [1]. It is also unclear whether trial or study registries need to be searched in all cases. While RCTs may be registered, registration of NRSI is far from standard. Fourth, it is unclear how SR completion is defined to assess if the search strategy was recent for the YES rating in the AMSTAR 2 tool [1]. The peer review process of a SR can take several months meaning that the SR can be published long after the actual completion of the SR. If there are more than 24 months between the last search and publication, the SR may only obtain a PARTIAL YES rating, although it was completed within 24 months. Fifth, including or consulting content experts needs to be done by default according to the AMSTAR 2 guidance document. However, it is unclear how experts are defined, how many should be consulted or whether they belong to the SR team or are external people not involved in SR production. An example of an expert could be a librarian or information specialist, who may also be a co-author on the SR.

### Item 5. Did the review authors perform study selection in duplicate? [1] (ratings: YES, NO)

There are two challenges with this item. First, the YES rating requires that studies were selected in duplicate and that high consensus was achieved between SR authors. It is unclear how to rate this item if consensus between SR authors is not mentioned. Second, additional explanations are required to rate this item. For example, according to the AMSTAR 2 tool [1] it is not clear whether the complete study selection process should be done in duplicate (i.e., screening of titles and abstracts as well as screening of full-texts), while the AMSTAR 2 guidance document [1] suggests that this may be required. Furthermore, the procedure for computing agreement or dealing with poor agreement between SR authors is not specified. For example, it is unclear if agreement should be computed for the complete study selection process and what sample size of studies should be chosen to compute agreement.

### Item 6. Did the review authors perform data extraction in duplicate? [1] (ratings: YES, NO)

Similar challenges as for item 5 also apply to this item. For example, it is not clear how to deal with small samples of extracted studies. In addition, this item refers only to data extraction and not to the risk of bias assessment of the primary studies. Thus, it is unclear if such assessment should also be performed in duplicate. This issue is neither covered by this item nor by item 9 (risk of bias in individual studies) on AMSTAR 2. Interestingly, the current version of the Cochrane Handbook suggests that it should be mandatory to perform the risk of bias assessment in duplicate (Version 6.3, Chap. 7, Sect. 7.3.2) [17].

### Item 7. Did the review authors provide a list of excluded studies and justify the exclusions? [1] (ratings: YES, PARTIAL YES, NO)

This item is particularly important for replicability of SRs and detecting any biases in study selection. There are two challenges with this item. First, it is unclear if the list of excluded studies should show all studies selected for full-text screening from title and abstract screening. The requirement for the PARTIAL YES rating indeed appears to refer to such studies from title and abstract screening as “all potentially relevant studies”. Second, it is unclear if reasons for exclusion should be reported only for studies screened in full-text or for all studies. Once again, it appears that the requirement for the YES rating refers only to studies screened in full-text as “each potentially relevant study”.

### Item 8. Did the review authors describe the included studies in adequate detail? [1] (ratings: YES, PARTIAL YES, NO)

This item requires that a SR describes PICO and study designs for the PARTIAL YES rating as well as setting and timeframe for follow-up for the YES rating. The majority of SRs indeed meet the minimum requirements for this item, resulting in a high proportion of PARTIAL YES ratings [13, 14]. However, the challenge with this item is that there are no thresholds for deciding if study characteristics are reported in adequate detail. Deciding whether information on study characteristics is adequately “detailed” is often judged differently among AMSTAR 2 users and requires a high degree of judgment and subjective decision making. According to the AMSTAR 2 guidance document [1] the details “should be sufficient for an appraiser, or user, to make judgments about the extent to which the studies were appropriately chosen (in relation to the PICO structure)”. Thus, the sufficient details may depend on SR aims as well as the individual expectations of AMSTAR 2 users.

### Item 9. Did the review authors use a satisfactory technique for assessing the risk of bias (RoB) in individual studies that were included in the review? [1] (separate ratings for RCTs and NRSI: YES, PARTIAL YES, NO, INCLUDES ONLY NRSI, INCLUDES ONLY RCTs)

This item distinguishes between RCTs and NRSIs and is accompanied by extensive notes for rating of NRSIs in the AMSTAR 2 guidance document [1]. There are two challenges with this item. First, it is unclear how to rate this item if the RoB assessment was adequately assessed in only one study type, such as RCTs, but not in NRSI or vice versa. Second, it is not explicitly required that the RoB should be assessed in duplicate. Such procedure is already recommended in the Cochrane handbook (Version 6.3, Chap. 7, Sect. 7.3.2) [17] and could reduce any difficulties in rating of this item.

### Item 10. Did the review authors report on the sources of funding for the studies included in the review? [1] (ratings: YES, NO)

Funding for primary studies and potential sources of conflict of interest in the SRs are addressed by two AMSTAR 2 items (items 10 and 16, respectively [1]). In general, the sources of funding in primary studies are often not reported in SRs and thus this item is not fulfilled by the vast majority of SRs [10, 14]. According to the AMSTAR 2 guidance document [1], the information on funding in item 10 is needed to assess any conflicts of interest related to such funding (e.g., bias towards results that favour products sponsored by study funders). The challenge with item 10 is that it addresses only the sources of funding in primary studies unlike item 16 that addresses any sources of conflict of interest (including funding for conducting the SR). Thus, the discrepancy between the content of both items creates a misconception that funding alone could affect primary studies while SRs could also have other sources of potential conflicts of interest. As stated in item 16 and in the AMSTAR 2 guidance document [1], there could be several sources of potential conflict of interest, such as professional conflicts.

### Item 11. If meta-analysis was performed did the review authors use appropriate methods for statistical combination of results? [1] (separate ratings for RCTs and NRSI: YES, NO, NO META-ANALYSIS CONDUCTED)

The AMSTAR 2 guidance document [1] lists some requirements for rating of the appropriateness of a meta-analysis. There are three challenges with this item. First, in contrast to the AMSTAR 2 guidance document [1], item 11 does not mention that the justification for performing meta-analysis should be planned a priori and included in the SR protocol. It is also not clear what information should be provided in such a justification, although the AMSTAR 2 guidance document [1] suggests that studies should be “compatible (in terms of populations, controls and interventions)”. Second, in addition to justification for meta-analysis, the YES rating has several other requirements that are in general not agreed upon in the field of meta-analysis, such as the type of weighting technique and computation or adjustment for heterogeneity. It is unclear how to rate this item if at least one of the requirements is not fulfilled. There is also no guidance on what specific meta-analytic methods can be rated as appropriate. In general, this item can be rated only if SRs provide detailed description of meta-analysis that is required to replicate the analysis and is in accordance with the Cochrane handbook (Version 6.3, Chap. 10: Analyzing data and undertaking meta-analyses) [17]. Such description should include (1) the effect-size computation, (2) weighting technique method (e.g., inverse-variance, Mantel-Haenszel), (3) statistical software package used, (4) meta-analytical model type for the main analysis (e.g., random-effects model), (5) computation of adjustment for heterogeneity, (6) exploration of heterogeneity, such as subgroup analysis, sensitivity analysis or meta-regression. Third, similar to item 9, it is unclear how to rate this item if meta-analysis was adequately performed in only one study type, such as RCTs, but not in NRSIs or vice versa.

### Item 12. If meta-analysis was performed, did the review authors assess the potential impact of RoB in individual studies on the results of the meta-analysis or other evidence synthesis? [1] (ratings: YES, NO, NO META-ANALYSIS CONDUCTED)

There are five challenges with this item. First, it is unclear how to rate this item because the AMSTAR 2 tool [1] refers to SRs with meta-analysis while the AMSTAR 2 guidance document [1] suggests that in the absence of meta-analysis SR authors “should still provide some commentary on the likely impact of RoB on individual study results”. Second, the item is fulfilled in case of low RoB, but only if SR includes RCTs, while no rating guidance is provided if SR includes NRSI with low RoB. Third, the item requires that SR authors perform sensitivity analyses to investigate the impact of variable or high RoB on the pooled effects in meta-analysis. However, it is unclear what analysis can be considered as adequate (e.g., meta-regression or subgroup analysis). Fourth, the rating of this item is difficult if sensitivity analysis is not performed because all studies in SR have a high RoB or there are too few studies for such an analysis. Fifth, it is unclear how to rate this item if the RoB assessment was already inadequately performed in item 9 and thus produced a biased RoB assessment.

### Item 13. Did the review authors account for RoB in individual studies when interpreting/discussing the results of the review? [1] (ratings: YES, NO)

Assuming that the RoB was adequately performed in item 9, the main challenge with rating of this item is to decide to what extent the impact of the RoB in primary studies should be discussed in a SR (e.g., one general sentence vs. a detailed paragraph on RoB of the primary and secondary outcomes). The AMSTAR 2 guidance document [1] notes that RoB should especially be considered in any recommendations for the clinical care or policy in a SR. It is also unclear how to rate this item if multiple outcomes were assessed in a SR, but the impact of RoB was discussed for example only for the primary outcome.

### Item 14. Did the review authors provide a satisfactory explanation for, and discussion of, any heterogeneity observed in the results of the review? [1] (ratings: YES, NO)

There are two challenges with this item. First, it is unclear how heterogeneity is defined in this item. According to the Cochrane Handbook, clinical and methodological heterogeneity can lead to a variation in study effect estimates that can be detected as statistical heterogeneity in a meta-analysis (Version 6.3, Chap. 10, Sect. 10.10.1) [17]. Clinical heterogeneity in terms of similar PICO factors must be considered in the justification for performing meta-analysis and in choosing the meta-analytic model (fixed-effect or random-effects). Statistical heterogeneity is often referred to simply as heterogeneity and can be measured in a meta-analysis. It appears that the term heterogeneity in this item refers to any heterogeneity (clinical, methodological or statistical). Second, it is unclear how heterogeneity should be computed and when the explanation and discussion of heterogeneity are adequate. In general, any sensitivity analyses, such as subgroup or meta-regression analyses require a meaningful number of studies (e.g., at least five). This number may not be reached if meta-analysis was performed with less than five studies (e.g., Cochrane Handbook, Version 6.3, Chap. 10, Sect. 10.10.2 [17]). Numerous and not preplanned subgroup analyses are questionable and affect the credibility of results of meta-analysis because of the increased risk for false-positive findings [18].

### Item 15. If they performed quantitative synthesis did the review authors carry out an adequate investigation of publication bias (small study bias) and discuss its likely impact on the results of the review? [1] (ratings: YES, NO, NO META-ANALYSIS CONDUCTED)

Publication bias can affect the outcomes of a meta-analysis and thus needs to be addressed in SRs along other biases in primary study selection or methods. There are five challenges with this item. First, it is unclear what investigation of publication bias can be considered adequate and what reasons for not performing the analysis (e.g., due to a small number of studies) are acceptable for the YES rating. Second, the requirement for the YES rating consists of two criteria: (1) that a publication bias test is performed and (2) that the result of such a test is discussed. However, it is unclear to what extent the results of a publication bias test should be discussed. Interestingly, this item also does not require that the publication bias test is (appropriately) reported in SRs. In fact, SR readers are unable to verify the interpretation of any potential publication bias if the results of a publication bias test are not reported in text or on a standard figure, such as a funnel plot. Third, publication bias cannot always be captured graphically or statistically in a meaningful way when less than 10 studies are included in a meta-analysis (e.g., Cochrane Handbook, Version 6.3, Chap. 13, Sect. 13.3.5.4 [17]). Interestingly, the AMSTAR 2 guidance document [1] highlights the importance of the context and setting of a SR (e.g., industry-sponsored SRs) as well as a deep and intensive literature search that includes, for example, the search for gray literature (see also item 4). Thus, in a methodological study, this item was pragmatically rated YES if the search strategy considered gray literature [14]. Fourth, the rating NO META-ANALYSIS CONDUCTED implies that publication bias is relevant only for SRs with meta-analysis. However, publication bias should be discussed as a potential source of bias in all SRs, albeit the extent of such discussion is not defined in this item. For example, one sentence in the limitations section may not be sufficient to address the publication bias in SR. Fifth, it is unclear if publication bias should be assessed and discussed for (1) each outcome in a meta-analysis separately and (2) for all studies in a meta-analysis irrespective of study types (RCTs and NRSI).

### Item 16. Did the review authors report any potential sources of conflict of interest, including any funding they received for conducting the review? [1] (ratings: YES, NO)

The challenge with item 16 is that it is not clear what sources of conflict of interest should be considered in SRs. While financial interests need to be disclosed in academic journals, there are also intellectual or institutional conflicts of interest that may apply to all or individual SR authors. Furthermore, it is unclear what constitutes an acceptable management of any potential conflicts. One suggestion could be that SR authors with potential conflicts of interest are not involved in some aspects of SR production (e.g., the risk of bias assessment of own studies included in SRs or overviews of SRs) and that they explicitly state any methods implemented to reduce the risk of any author biases [19, 20].

## Other challenges with AMSTAR 2 use

The appraisal outcome on AMSTAR 2 is the overall confidence rating in the results of a SR (high, moderate, low, or critically low) [1]. The confidence ratings can be derived based on a combination of ratings on individual AMSTAR 2 items (Box 1 in [1]). In general, the 16 items on AMSTAR 2 consist of seven critical items and nine non-critical items. There are four challenges for deriving the overall confidence ratings on AMSTAR 2. First, it is unclear how to interpret the PARTIAL YES ratings, as already mentioned elsewhere [21]. The rating algorithm (Box 1 in [1]) refers to the presence or absence of weaknesses on critical or non-critical items. Any weakness can lead to downgrading of the overall confidence rating. Thus, if any PARTIAL YES ratings are judged as weaknesses then the overall confidence rating may be downgraded (e.g., from high to moderate). Second, it is unclear how many non-critical weaknesses are required to downgrade the confidence ratings from moderate to low. Third, AMSTAR 2 users can decide to modify the list of critical items for the overall confidence rating according to their specific research question. However, there is no guidance for deciding which items can be included or omitted from such a list. AMSTAR 2 users should transparently describe and justify the use of an alternative list of critical items such that AMSTAR 2 ratings could be compared among studies. Fourth, it is unclear what rating algorithm is incorporated in the online version of AMSTAR 2 (www.amstar.ca) as there are inconsistencies with the ratings derived from the AMSTAR 2 tool [22].

## Summary of recommendations for AMSTAR 2 users

We identified various challenges with the content of 15/16 items on AMSTAR 2. Based on these challenges we formulated recommendations for AMSTAR 2 users (Table 1). These challenges could be addressed by additional decision rules including definitions for ambiguous items and guidance for rating of complex items and derivation of confidence ratings. We recommend that a team consensus regarding such decision rules is required before appraisal procedure begins. These recommendations could ease the appraisal procedure and improve the consensus in rating among a team of AMSTAR 2 users. In general, while appraisal can be performed by one author alone, appraising SRs in a team could improve the interrater reliability of ratings and the general usability of the tool. Any additional decision rules need to be piloted and transparently described to improve the reproducibility of ratings.

**Table 1:** Recommendations for AMSTAR 2 users

Item or overall rating	Recommendation
1. Components of PICO	-
**2. Review protocol** ^**a**^	• Rating requires access to SR protocol • Team consensus is required to decide how to rate this item if (1) major deviations from registered SR protocol exist (including a definition of ‘major deviations’) and (2) SR protocol is registered shortly before submission of the completed SR
3. Selection of study designs	• Rating requires that justification for selection of specific study designs is either inferred from SR text (e.g., rationale for SR) or explained by SR authors
**4. Comprehensive literature search** ^**a**^	• Rating requires access to full search strategy • Team consensus is required to define (1) information that should be included in the full strategy, (2) how to deal with Cochrane SR that search only one Cochrane database, (3) how to deal with gray literature and if trial registries should be searched, (4) SR completion date, (5) experts relevant for the SR
5. Study selection in duplicate	• Team consensus is required to decide (1) how to rate this item if low consensus exists between SR authors, (2) the requirements for computing agreement between SR authors and deal with poor agreement
6. Data extraction in duplicate	• Team consensus is required to decide if risk of bias assessment in primary studies should be performed in duplicate
**7. List of excluded studies** ^**a**^	• Rating requires that excluded studies are reported in SR (as a list, table or in text of SR or its supplementary materials) • Team consensus is required to define potentially relevant studies for SR
8. Adequate study characteristics	• Team consensus is required to decide what type of information will be considered as adequately detailed
**9. Satisfactory technique for assessing RoB** ^**a**^	• Team consensus is required to decide how to rate this item if (1) different ratings exist for RCTs and NRSIs in the same SR (e.g., choose the more conservative rating as the overall item rating), or (2) the risk of bias assessment was performed by a single SR author
10. Sources of funding for primary studies	• Team consensus is required to decide how to deal with a report of potential conflict of interest other than the source of funding in the primary studies in SR
**11. Appropriate methods of meta-analysis** ^**a**^	• Rating requires detailed description of meta-analysis • Team consensus is required to decide (1) if adequate information was provided to justify and to replicate meta-analysis, and (2) how to rate this item if different ratings exist for RCTs and NRSIs in the same SR (e.g., choose the more conservative rating as the overall item rating)
12. Impact of RoB on the results assessed	• Team consensus is required to (1) decide if the RoB impact should be discussed in the absence of meta-analysis, (2) decide how to rate this item if SR includes NRSI with low RoB, (3) define what sensitivity analysis is adequate, (4) decide how to rate this item if sensitivity analysis cannot be performed due to the same (high) RoB in all studies or too small number of studies, and (5) decide how to rate this item if RoB was inadequately performed in item 9
**13. Impact of RoB on the results discussed** ^**a**^	• Team consensus is required to decide (1) to what extent the impact of the RoB in primary studies should be discussed in a SR and (2) how to rate this item if multiple outcomes were assessed in a SR, but the impact of RoB was discussed only for the primary outcome
14. Explanation and discussion of heterogeneity	• Rating requires additional information about heterogeneity (e.g., computation method and preplanning any sensitivity analyses) • Team consensus is required to (1) define heterogeneity and (2) decide how heterogeneity should be computed and when the explanation and discussion of heterogeneity are adequate
**15. Publication bias assessed and discussed** ^**a**^	• Rating requires that the outcomes of publication bias test should be reported in SR either in text or on a standard figure, such as a funnel plot • Team consensus is required to decide (1) if investigation of publication bias is adequate, (2) if discussion of publication bias is adequate, (3) if reasons for not performing a publication bias test are explained, (4) if publication bias was addressed in SR without meta-analysis, (5) if publication bias should be assessed for all studies or for each outcome or for each study design in SR
16. Sources of funding for review	• Team consensus is required to decide how to define (1) the sources of conflict of interest in SR and (2) acceptable management of such conflicts in SR
**Overall confidence rating**	• Team consensus is required to (1) decide if PARTIAL YES ratings are critical weaknesses, (2) define how many non-critical weaknesses are required to downgrade the confidence ratings from moderate to low, (3) define an alternative list of critical items

Note. ^a^Critical items for the overall confidence rating in the results of SR as suggested by AMSTAR 2 developers [1]. Abbreviations: AMSTAR 2, A Measurement Instrument to Assess Systematic Reviews, version 2; NRSI, non-randomized studies of interventions; PICO, Population, Intervention, Comparator, Outcome; RCT, randomized-controlled trial; RoB, risk of bias; SR, systematic review

## Comparison with the relevant literature

The work on this commentary was motivated by the results of overviews or meta-research studies and our own experience with AMSTAR 2 use. In general, AMSTAR 2 produces a high proportion of critically low confidence ratings for SRs of health interventions in various clinical fields (for example, see [10, 11, 13, 14, 23–28]). There is an ongoing debate about whether the cause of such critically low confidence ratings is the poor discriminatory power of the tool or the low quality of SRs of healthcare interventions [10, 11, 24, 25]. This debate was not addressed in the context of our commentary and requires further investigation.

In this commentary we focus on challenges with the use of the AMSTAR 2 tool from the user´s perspective. In fact, a similar approach was used following the publication of the original version of AMSTAR [2]. Various user groups commented on the challenges with the tool content [29–31] and the limited guidance for users [32, 33]. In general, additional decision rules for users were recommended to address the challenges with AMSTAR [33]. Such user feedback described in these articles was used to develop AMSTAR 2 [1]. Specifically, AMSTAR items were revised and new items were added to enable appraisal of SRs of RCTs and NRSIs, to better focus on methodological than mere reporting quality and to include new requirements for SRs (e.g., protocol registration). Furthermore, the rating of the confidence in the results of SRs was introduced based on weaknesses on critical and non-critical items.

Our commentary is the first in-depth examination of the AMSTAR 2 tool from the user perspective. We add to the first reports of difficulties with AMSTAR 2 by other users in different clinical fields. Perry et al. [6] noted that AMSTAR 2 was in general user-friendly, but more response options on some items could improve its usability. Leclercq et al. [10] identified items 11 and 14 as not precise enough for consistent rating of 206 SRs with meta-analysis. Li et al. [11] suggested that AMSTAR 2 may be too unreasonable in terms of item rating and derivation of confidence ratings. Their appraisals of 81 SRs showed that despite being published in high impact journals, most SRs obtained critically low ratings. The authors criticized the all-or-none rating of items (as YES or NO) that does not allow to differentiate between missing information and incomplete reporting of information on some items [11]. They also questioned if critically low confidence should be assigned to SRs without protocols and without a list of excluded studies that were identified as the items responsible for the critically low ratings in their study [11], but also in other studies (for example, see [10, 13, 25]). Two studies ([10, 11]) noted the apparent floor effect (i.e., the overestimation of critically low confidence ratings) that was due to the choice of critical and non-critical items on AMSTAR 2. Furthermore, Dijkers et al. [9] noted that extended discussions and leniency in rating were required to appraise 17 SRs, while Hou et al. [34] mentioned that the rating of some items was subjective and potentially leading to bias in ratings of 11 SRs. To address these first criticisms of AMSTAR 2 and similar to the critique of AMSTAR [33], we recommend that additional decision rules should be considered and team consensus on these rules is required before the appraisal procedure begins. Our recommendations aim to initiate a discussion of what could be done better by the users to improve the usability of the tool. Our commentary could also be considered by AMSTAR 2 developers to further revise this frequently used tool.

## Limitations

There were several limitations in this commentary. First, we provide recommendations for AMSTAR 2 users in addition to already existing and extensive user guidance document [1]. Rather than to potentially overwhelm the users with additional guidance, our recommendations could be considered if the AMSTAR 2 items or the guidance document are inadequate in a specific field. Second, our approach to identifying the challenges was descriptive and we reached consensus during discussion. Thus, further studies using focus group, Delphi or quantitative survey methodology could be used to systematically assess the challenges with AMSTAR 2 according to its users. Third, this commentary is based on opinions of a small group of methodologists. Although we are experienced users of AMSTAR 2 in different clinical fields, a debate among a wider group of researchers could detect further challenges with the tool. For example, detecting the PICO elements anywhere in the SR (required to rate item 1 on AMSTAR 2) was not perceived as challenging by us, while other AMSTAR 2 users noted difficulties in rating this item [9]. Thus, other researchers may recommend additional guidelines to detect PICO or that PICO should be stated in study abstracts because explicit use of PICO elements in abstracts facilitates the search, retrieval, screening and coding of the relevant studies in SRs [35].

## Future directions

Our recommendations for rating of 15/16 AMSTAR 2 items need to be empirically tested to investigate if they could ease the appraisal procedure and improve the consensus in rating among AMSTAR 2 users. AMSTAR 2 developers could also consider these recommendations to further improve the usability of the tool.

## Implications for practice

There is an exponential increase in the number of published SRs of healthcare interventions [36, 37]. Thus, evidence appraisal tools for SRs, such as AMSTAR 2, are increasingly more important to evaluate if the SRs are methodologically sound and designed to reduce bias in their outcomes [38]. AMSTAR 2 could be used to detect SRs with the lowest confidence ratings and exclude them from the pool of relevant SRs for policy development or health decision-making [39]. Such classification of SRs could be helpful for decision makers, who find it difficult to select appropriate SRs for their work [40]. Our recommendations could be considered to improve the usability of AMSTAR 2 for health decision-making.

## Summary

Our commentary adds to the existing literature by providing the first in-depth examination of the AMSTAR 2 tool from the user perspective. Although AMSTAR 2 is a validated and popular tool for SR appraisals, our commentary shows that various challenges exist with the tool based on the perspective of six experienced users. The identified challenges could be addressed by additional decision rules including definitions for ambiguous items and guidance for rating of complex items and derivation of confidence ratings. We recommend that a team consensus regarding such decision rules is required before appraisal procedure begins.

## Data Availability

All data generated or analysed are included in this article.

## Abbreviations

AMSTAR 2: A Measurement Tool to Assess Systematic Reviews
NRSI: non-randomized studies of interventions
PICO: Population, Intervention, Comparator, Outcome
RCT: randomized-controlled trial
RoB: risk of bias
ROBIS: Risk of Bias in Systematic Reviews
SR: systematic review

## References

[1] Shea BJ, Reeves BC, Wells G, Thuku M, Hamel C, Moran J, Moher D, Tugwell P, Welch V, Kristjansson E (2017). AMSTAR 2: a critical appraisal tool for systematic reviews that include randomised or non-randomised studies of healthcare interventions, or both. BMJ.

[2] Shea BJ, Grimshaw JM, Wells GA, Boers M, Andersson N, Hamel C, Porter AC, Tugwell P, Moher D, Bouter LM (2007). Development of AMSTAR: a measurement tool to assess the methodological quality of systematic reviews. BMC Med Res Methodol.

[3] Whiting P, Savović J, Higgins JPT, Caldwell DM, Reeves BC, Shea B, Davies P, Kleijnen J, Churchill R, Group R (2016). ROBIS: a new tool to assess risk of bias in systematic reviews was developed. J Clin Epidemiol.

[4] Lorenz RC, Matthias K, Pieper D, Wegewitz U, Morche J, Nocon M, Rissling O, Schirm J, Jacobs A (2019). A psychometric study found AMSTAR 2 to be a valid and moderately reliable appraisal tool. J Clin Epidemiol.

[5] Pieper D, Puljak L, González-Lorenzo M, Minozzi S (2019). Minor differences were found between AMSTAR 2 and ROBIS in the assessment of systematic reviews including both randomized and nonrandomized studies. J Clin Epidemiol.

[6] Perry R, Whitmarsh A, Leach V, Davies P (2021). A comparison of two assessment tools used in overviews of systematic reviews: ROBIS versus AMSTAR-2. Syst Rev.

[7] Gates M, Gates A, Duarte G, Cary M, Becker M, Prediger B, Vandermeer B, Fernandes RM, Pieper D, Hartling L (2020). Quality and risk of bias appraisals of systematic reviews are inconsistent across reviewers and centers. J Clin Epidemiol.

[8] Leclercq V, Beaudart C, Tirelli E, Bruyère O (2020). Psychometric measurements of AMSTAR 2 in a sample of meta-analyses indexed in PsycINFO. J Clin Epidemiol.

[9] Dijkers MP, Akers KG, Dieffenbach S, Galen SS (2021). Systematic reviews of clinical benefits of Exoskeleton Use for Gait and mobility in neurologic Disorders: a tertiary study. Arch Phys Med Rehabil.

[10] Leclercq V, Beaudart C, Ajamieh S, Tirelli E, Bruyère O (2020). Methodological quality of meta-analyses indexed in PsycINFO: leads for enhancements: a meta-epidemiological study. BMJ Open.

[11] Li L, Asemota I, Liu B, Gomez-Valencia J, Lin L, Arif AW, Siddiqi TJ, Usman MS (2022). AMSTAR 2 appraisal of systematic reviews and meta-analyses in the field of heart failure from high-impact journals. Syst Rev.

[12] Lucchetta RC, Leonart LP, Gonçalves MVM, Becker J, Pontarolo R, Fernandez-Llimós F, Wiens A (2020). Reliability in long-term clinical studies of disease-modifying therapies for relapsing-remitting multiple sclerosis: a systematic review. PLoS ONE.

[13] Matthias K, Rissling O, Pieper D, Morche J, Nocon M, Jacobs A, Wegewitz U, Schirm J, Lorenz RC (2020). The methodological quality of systematic reviews on the treatment of adult major depression needs improvement according to AMSTAR 2: a cross-sectional study. Heliyon.

[14] Siemens W, Schwarzer G, Rohe MS, Buroh S, Meerpohl JJ, Becker G (2021). Methodological quality was critically low in 9/10 systematic reviews in advanced cancer patients-A methodological study. J Clin Epidemiol.

[15] Almeida MO, Yamato TP, Parreira P, Costa LOP, Kamper S, Saragiotto BT (2020). Overall confidence in the results of systematic reviews on exercise therapy for chronic low back pain: a cross-sectional analysis using the assessing the Methodological Quality of systematic reviews (AMSTAR) 2 tool. Braz J Phys Ther.

[16] Shokraneh F, Adams CE. Cochrane Schizophrenia Group’s study-based Register of Randomized controlled trials: development and content analysis. Schizophr Bull Open. 2020;1(1). 10.1093/schizbullopen/sgaa061

[17] Higgins JPTTJ, Chandler J, Cumpston M, Li T, Page MJ, Welch VA, editors, editors. Cochrane Handbook for Systematic Reviews of Interventions (version 6.3 updated February 2022). *Cochrane, Available from wwwtrainingcochraneorg/handbook*

[18] Schandelmaier S, Briel M, Varadhan R, Schmid CH, Devasenapathy N, Hayward RA, Gagnier J, Borenstein M, van der Heijden G, Dahabreh IJ (2020). Development of the instrument to assess the credibility of Effect modification analyses (ICEMAN) in randomized controlled trials and meta-analyses. CMAJ.

[19] Aromataris E. Compounding conflicts of interest: including an author’s own work in a systematic review. JBI Evid Synth. 2022;20(8). 10.11124/JBIES-22-00272 10.11124/JBIES-22-00272 35971197

[20] Pieper D, Waltering A, Holstiege J, Büchter RB (2018). Quality ratings of reviews in overviews: a comparison of reviews with and without dual (co-)authorship. Syst Rev.

[21] Lorenz RC, Pieper D, Rombey T, Jacobs A, Rissling O, Freitag S, Matthias K: Reply to letter to the editor by, Franco et al. AMSTAR 2 overall confidence rating: A call for even more transparency. *J Clin Epidemiol* 2021, 138:241–242. 10.1016/j.jclinepi.2021.03.016 10.1016/j.jclinepi.2021.03.016 33771573

[22] Pieper D, Lorenz RC, Rombey T, Jacobs A, Rissling O, Freitag S, Matthias K (2021). Authors should clearly report how they derived the overall rating when applying AMSTAR 2-a cross-sectional study. J Clin Epidemiol.

[23] Antony J, Brar R, Khan PA, Ghassemi M, Nincic V, Sharpe JP, Straus SE, Tricco AC (2020). Interventions for the prevention and management of occupational stress injury in first responders: a rapid overview of reviews. Syst Rev.

[24] Lorenz RC, Matthias K, Pieper D, Wegewitz U, Morche J, Nocon M, Rissling O, Schirm J, Freitag S, Jacobs A (2020). AMSTAR 2 overall confidence rating: lacking discriminating capacity or requirement of high methodological quality?. J Clin Epidemiol.

[25] De Santis KK, Lorenz RC, Lakeberg M, Matthias K (2022). The application of AMSTAR2 in 32 overviews of systematic reviews of interventions for mental and behavioural disorders: a cross-sectional study. Res Synth Methods.

[26] Gold N, Yau A, Rigby B, Dyke C, Remfry EA, Chadborn T (2021). Effectiveness of Digital Interventions for reducing behavioral risks of Cardiovascular Disease in Nonclinical Adult populations: systematic review of reviews. J Med Internet Res.

[27] Kracht CL, Hutchesson M, Ahmed M, Müller AM, Ashton LM, Brown HM, DeSmet A, Maher CA, Mauch CE, Vandelanotte C (2021). E-&mHealth interventions targeting nutrition, physical activity, sedentary behavior, and/or obesity among children: a scoping review of systematic reviews and meta-analyses. Obes Rev.

[28] Motahari-Nezhad H, Al-Abdulkarim H, Fgaier M, Abid MM, Péntek M, Gulácsi L, Zrubka Z (2022). Digital Biomarker-Based interventions: systematic review of systematic reviews. J Med Internet Res.

[29] Burda BU, Holmer HK, Norris SL (2016). Limitations of a Measurement Tool to assess systematic reviews (AMSTAR) and suggestions for improvement. Syst Rev.

[30] Faggion CM (2015). Critical appraisal of AMSTAR: challenges, limitations, and potential solutions from the perspective of an assessor. BMC Med Res Methodol.

[31] Wegewitz U, Weikert B, Fishta A, Jacobs A, Pieper D (2016). Resuming the discussion of AMSTAR: what can (should) be made better?. BMC Med Res Methodol.

[32] Pieper D, Koensgen N, Breuing J, Ge L, Wegewitz U (2018). How is AMSTAR applied by authors - a call for better reporting. BMC Med Res Methodol.

[33] Pollock M, Fernandes RM, Hartling L (2017). Evaluation of AMSTAR to assess the methodological quality of systematic reviews in overviews of reviews of healthcare interventions. BMC Med Res Methodol.

[34] Hou T, Zheng Q, Feng X, Wang L, Liu Y, Li Y (2020). Methodology and reporting quality evaluation of acupuncture for mild cognitive impairment: an overview of systematic reviews. Evid Based Complement Alternat Med.

[35] Brockmeier AJ, Ju M, Przybyła P, Ananiadou S (2019). Improving reference prioritisation with PICO recognition. BMC Med Inform Decis Mak.

[36] Breuer C, Meerpohl JJ, Siemens W (2023). From standard systematic reviews to living systematic reviews. Z Evid Fortbild Qual Gesundhwes.

[37] Hoffmann F, Allers K, Rombey T, Helbach J, Hoffmann A, Mathes T, Pieper D (2021). Nearly 80 systematic reviews were published each day: observational study on trends in epidemiology and reporting over the years 2000–2019. J Clin Epidemiol.

[38] Heise TL, Seidler A, Girbig M, Freiberg A, Alayli A, Fischer M, Haß W, Zeeb H (2022). CAT HPPR: a critical appraisal tool to assess the quality of systematic, rapid, and scoping reviews investigating interventions in health promotion and prevention. BMC Med Res Methodol.

[39] De Santis K, Matthias K. Different approaches to appraising systematic reviews of digital interventions for physical activity promotion using AMSTAR 2 tool: Cross-sectional study. Int J Environ Res Public Health. 2023; 20(6), 4689; 10.3390/ijerph20064689 10.3390/ijerph20064689 PMC10048476 36981598

[40] Lunny C, Whitelaw S, Chi Y, Zhang J, Ferri N, Pieper SK, Shea D, Dourka B, Veroniki J et al. A : Decision makers find it difficult to compare and select similar systematic reviews based on quality, methods and results: a cross-sectional survey. *Preprint (Version 1)* 10 January 2023. 10.21203/rs.3.rs-2416773/v1

